# Impact of the Project P.A.T.H.S. in the Junior Secondary School Years: Individual Growth Curve Analyses

**DOI:** 10.1100/tsw.2011.6

**Published:** 2011-02-03

**Authors:** Daniel T. L. Shek, Cecilia M. S. Ma

**Affiliations:** ^1^ Department of Applied Social Sciences, The Hong Kong Polytechnic University, Hong Kong; ^2^ Public Policy Research Institute, The Hong Kong Polytechnic University, Hong Kong; ^3^ Department of Sociology, East China Normal University, Shanghai, China; ^4^ Kiang Wu Nursing College of Macau, Macau, China; ^5^ Department of Pediatrics, University of Kentucky College of Medicine, Lexington, KY, USA

**Keywords:** Project P.A.T.H.S., positive youth development program, randomized group trial, individual growth curves

## Abstract

The Tier 1 Program of the Project P.A.T.H.S. (Positive Adolescent Training through Holistic Social Programs) is a positive youth development program implemented in school settings utilizing a curricular-based approach. In the third year of the Full Implementation Phase, 19 experimental schools (n = 3,006 students) and 24 control schools (n = 3,727 students) participated in a randomized group trial. Analyses based on linear mixed models via SPSS showed that participants in the experimental schools displayed better positive youth development than did participants in the control schools based on different indicators derived from the Chinese Positive Youth Development Scale, including positive self-identity, prosocial behavior, and general positive youth development attributes. Differences between experimental and control participants were also found when students who joined the Tier 1 Program and perceived the program to be beneficial were employed as participants of the experimental schools. The present findings strongly suggest that the Project P.A.T.H.S. is making an important positive impact for junior secondary school students in Hong Kong.

## INTRODUCTION

The Project P.A.T.H.S. (**P**ositive **A**dolescent **T**raining through **H**olistic **S**ocial Programs) is a youth enhancement program that attempts to promote holistic youth development in Hong Kong[1,2]. There are two tiers of programs (Tier 1 and Tier 2) in this project. The Tier 1 Program is a universal positive youth development program where students in Secondary 1 to 3 normally participate in a 20-h program in the school year at each grade. According to Catalano et al.[3], several positive youth development aspects are important. These include promotion of bonding, cultivation of resilience, promotion of social competence, promotion of emotional competence, promotion of cognitive competence, promotion of behavioral competence, promotion of moral competence, cultivation of self-determination, promotion of spirituality, development of self-efficacy, development of a clear and positive identity, promotion of beliefs in the future, provision of recognition for positive behavior, provision of opportunities for prosocial involvement, and fostering prosocial norms. To help adolescents develop in a holistic manner, these 15 adolescent developmental constructs are covered in the project, particularly in the Tier 1 Program. The conceptual model of the project can be seen in Shek[2].

One unique characteristic of the Project P.A.T.H.S. is the use of a systematic and ongoing evaluation approach that monitors various aspects of the program. Utilizing the principle of triangulation, a wide range of evaluation strategies is used to evaluate the Tier 1 Program as follows:

1. Objective Outcome Evaluation: A randomized group trial with 24 experimental schools and 24 control schools initially was carried out.
2. Subjective Outcome Evaluation (Tier 1 Program): Both students and program implementers were invited to complete subjective outcome evaluation forms (Form A and Form B, respectively) after completion of the program.
3. Process Evaluation: Systematic observations were carried out in randomly selected schools in order to understand the program implementation details.
4. Interim Evaluation: To understand the process of implementation, interim evaluation was conducted by randomly selecting roughly half of the participating schools in the Experimental and Full Implementation Phases.
5. Qualitative Evaluation (Focus Groups Based on Students): Focus groups involving students based on schools randomly selected from the participating schools were conducted.
6. Qualitative Evaluation (Focus Groups Based on Program Implementers): Focus groups involving instructors based on schools randomly selected from the participating schools were carried out.
7. Qualitative Evaluation (In-Depth Interviews with Program Implementers): Prolonged in-depth interviews with teachers were conducted.
8. Qualitative Evaluation (Case Study Based on Focus Groups): A case study documenting the implementation experience of schools that have incorporated the Tier 1 Program into school formal curriculum was carried out.
9. Qualitative Evaluation (Student Logs): Students were invited to reflect upon their experiences after attending P.A.T.H.S. lessons and application of things they learned in class to real life.
10. Qualitative Evaluation (Student Products): Students' weekly diaries were collected after completion of the program. Students' drawings were also collected to reflect the experiences of the program participants.
11. Management Information Collected from the Co-Walker Scheme: The information collected by the *co-walkers*, who conducted classroom observations and completed observation forms, could give an overall picture about the implementation details in different schools.
12. Evaluation Based on the Repertory Grid Tests: Students were randomly selected to complete repertory grid tests in order to assess their self-identity systems before and after joining the program and the perceived changes across years.

Generally speaking, triangulation of the available evaluation findings showed that different stakeholders had positive views toward the Tier 1 Program and perceived the program to be beneficial to the development of the program participants. Most importantly, the findings suggest that the project is effective in promoting positive youth development among Chinese adolescents in Hong Kong[4,5,6,7,8].

As far as objective outcome evaluation is concerned, several studies have shown that students who participated in the project showed better development than those who did not participate. Utilizing a pre-experimental design, Shek[9] showed that there were positive changes in the program participants in many measures of positive youth development. Based on the first two waves of data collected in a randomized group trial, Shek et al.[8] showed that participants in the experimental schools had significantly higher positive youth development levels than those in the control schools. By using the first four waves of data collected in the first 2 years of the Full Implementation Phase, analyses based on generalized linear models and linear mixed methods showed that students in the experimental schools generally developed better than those in the control schools[7,10].

In a recent study of the six waves of data, Shek and Sun[6] reported that the Project P.A.T.H.S. was effective in promoting positive development among Hong Kong young people. Analyses of covariance and linear mixed models revealed that participants in the experimental schools showed significantly better development than those in the control schools based on several indicators of positive youth development derived from the Chinese Positive Youth Development Scale and other measures. Although the above findings provide support for the effectiveness of the Tier 1 Program of the Project P.A.T.H.S., it is noteworthy that some advanced techniques, including hierarchical linear modeling and latent growth curve modeling, have been developed in the past few decades[11,12,13,14,15,16,17]. Among these methods, individual growth curve modeling (IGC), also known as hierarchical linear modeling and multilevel modeling, is commonly used by researchers to examine individual changes over time. In the present study, IGC based on SPSS was used primarily to examine the treatment effects on youth development over time.

## METHODS

### Participants and Procedures

Shek and associates[8] described the procedures and criteria for recruiting the initial 24 experimental schools (i.e., one school dropped out after Wave 1) and 24 control schools in Year 1, during which the Waves 1 and 2 data were collected from Secondary 1 students. In Year 2, Waves 3 and 4 data were collected from the same cohort promoted to Secondary 2, with 20 experimental schools (i.e., three schools withdrew after Wave 2) and 24 control schools. In Year 3, Waves 5 and 6 data were collected from the same cohort with 19 experimental schools (i.e., one experimental school dropped out after Wave 4) and 24 control schools. The number of completed questionnaires collected in each measurement occasion can be seen in Table 1.

**TABLE 1**
**Number of Participants at Each Measurement Occasion**

| | Wave 1 | Wave 2 | Wave 3 | Wave 4 | Wave 5 | Wave 6 |
|---|---|---|---|---|---|---|
| N (school) | 48 | 47[a] | 44[b] | 44 | 43[c] | 43 |
| No. of participants | 7,846 | 7,388 | 6,939 | 6,697 | 6,876 | 6,733 |
| Control group | 3,797 | 3,654 | 3,765 | 3,698 | 3,757 | 3,727 |
| Male | 1,936 | 1,876 | 1,896 | 1,888 | 1,874 | 1,894 |
| Female | 1,613 | 1,619 | 1,666 | 1,599 | 1,682 | 1,679 |
| Experimental group | 4,049 | 3,734 | 3,174 | 2,999 | 3,119 | 3,006 |
| Male | 2,154 | 1,998 | 1,691 | 1,548 | 1,632 | 1,591 |
| Female | 1,745 | 1,571 | 1,283 | 1,259 | 1,312 | 1,278 |
| % of successfully matched | 98% | 96% | 97% | 98% | 99% | 97% |

[a] One experimental school (n = 207) had withdrawn after Wave 1.

[b] Three experimental schools (n = 629) had withdrawn after Wave 2.

[c] One experimental school (n = 71) had withdrawn after Wave 4.

At pre- and post-test, the purpose of the study was mentioned, and confidentiality of the collected data was repeatedly emphasized to all students in attendance on the day of testing. Parental and student consent was obtained prior to data collection. All participants responded to all scales in the questionnaire in a self-administration format. Adequate time was provided for the participants to complete the questionnaire. A trained research assistant was present throughout the administration process.

## Instruments

Consistent with the procedures used in Year 1, the participants were invited to respond to a questionnaire that comprised different measures of youth development at pretest (i.e., before the program began) and post-test (i.e., after the program ended). The following measures were used.

### *Chinese Positive Youth Development Scale (CPYDS)*

Based on the analyses conducted in Year 1, the item composition of the 15 subscales of the CPYDS are as follows:

1. Bonding Subscale (six items)
2. Resilience Subscale (six items)
3. Social Competence Subscale (seven items)
4. Emotional Competence Subscale (six items)
5. Cognitive Competence Subscale (six items)
6. Behavioral Competence Subscale (modified five items)
7. Moral Competence Subscale (six items)
8. Self-Determination Subscale (five items)
9. Self-Efficacy Subscale (modified two items)
10. Beliefs in the Future Subscale (modified three items)
11. Clear and Positive Identity Subscale (seven items)
12. Spirituality Subscale (seven items)
13. Prosocial Involvement Subscale (five items)
14. Prosocial Norms Subscale (five items)
15. Recognition for Positive Behavior Subscale (four items)

As mentioned by Shek[2], different composite indices derived from the scale were used to assess positive youth development. First and foremost, according to Shek et al.[8], the mean of the total mean score based on 12 subscales (excluding behavioral competence, self-determination, and prosocial norms) could be used as an overall measure of positive youth development (CPYDS-12). Next, as it can be argued that constructs including spirituality, prosocial norms, prosocial involvement, bonding, and recognition for positive behavior are different from the rest of the scales, a summation of 10 subscales (CPYDS-10) assessing psychosocial competence and strengths was used (i.e., resilience, social competence, emotional competence, cognitive competence, behavioral competence, moral competence, self-determination, self-efficacy, beliefs about the future, and clear and positive identity). Third, based on conceptual analyses of the items, one key item was derived for each domain, which resulted in a 15-item key measure (KEY 15). Fourth, based on item analysis, a 36-item measure was derived (KEY 36). Shek and Ma[18] also showed that the 15 scales in the CPYDS could be further reduced to four dimensions, including cognitive-behavioral competencies (CBC), prosocial attributes (PA), positive identity (PID), and general positive youth development qualities (GPYDQ). In general, high scores of these variables suggested better positive youth development. The internal consistency of these measures can be seen in Table 2.

**TABLE 2**
**Internal Consistency and Mean Interitem Correlations for All Indicators of the CPYDS**

| | Wave 1 | | Wave 2 | | Wave 3 | | Wave 4 | | Wave 5 | | Wave 6 | |
|---|---|---|---|---|---|---|---|---|---|---|---|---|
| | α | Mean[a] | α | Mean[a] | α | Mean[a] | α | Mean[a] | α | Mean[a] | α | Mean[a] |
| BO | 0.83 | 0.45 | 0.85 | 0.49 | 0.86 | 0.51 | 0.88 | 0.54 | 0.88 | 0.55 | 0.88 | 0.55 |
| RE | 0.82 | 0.44 | 0.86 | 0.50 | 0.88 | 0.54 | 0.88 | 0.55 | 0.89 | 0.56 | 0.88 | 0.55 |
| SC | 0.83 | 0.42 | 0.86 | 0.47 | 0.87 | 0.51 | 0.87 | 0.50 | 0.89 | 0.53 | 0.88 | 0.52 |
| PB | 0.76 | 0.44 | 0.80 | 0.51 | 0.83 | 0.55 | 0.83 | 0.56 | 0.85 | 0.58 | 0.84 | 0.58 |
| EC | 0.83 | 0.44 | 0.85 | 0.48 | 0.86 | 0.51 | 0.86 | 0.51 | 0.87 | 0.52 | 0.86 | 0.51 |
| CC | 0.84 | 0.47 | 0.86 | 0.52 | 0.87 | 0.54 | 0.88 | 0.54 | 0.88 | 0.56 | 0.88 | 0.55 |
| BC | 0.76 | 0.38 | 0.80 | 0.44 | 0.82 | 0.47 | 0.82 | 0.48 | 0.83 | 0.49 | 0.83 | 0.50 |
| MC | 0.78 | 0.37 | 0.79 | 0.39 | 0.81 | 0.42 | 0.80 | 0.41 | 0.82 | 0.44 | 0.82 | 0.43 |
| SD | 0.76 | 0.40 | 0.80 | 0.44 | 0.82 | 0.48 | 0.81 | 0.47 | 0.82 | 0.47 | 0.82 | 0.48 |
| SE | 0.50 | 0.34 | 0.56 | 0.39 | 0.58 | 0.41 | 0.59 | 0.42 | 0.61 | 0.43 | 0.61 | 0.44 |
| CPI | 0.84 | 0.43 | 0.85 | 0.45 | 0.87 | 0.48 | 0.86 | 0.47 | 0.87 | 0.48 | 0.87 | 0.49 |
| BF | 0.82 | 0.61 | 0.83 | 0.62 | 0.84 | 0.64 | 0.84 | 0.65 | 0.85 | 0.66 | 0.84 | 0.65 |
| PI | 0.83 | 0.49 | 0.83 | 0.50 | 0.86 | 0.55 | 0.85 | 0.52 | 0.86 | 0.55 | 0.86 | 0.54 |
| PN | 0.77 | 0.40 | 0.80 | 0.45 | 0.81 | 0.46 | 0.81 | 0.46 | 0.81 | 0.46 | 0.81 | 0.47 |
| SP | 0.88 | 0.51 | 0.89 | 0.56 | 0.91 | 0.60 | 0.91 | 0.60 | 0.92 | 0.62 | 0.91 | 0.62 |
| KEY 15 | 0.88 | 0.32 | 0.89 | 0.35 | 0.90 | 0.38 | 0.90 | 0.37 | 0.90 | 0.39 | 0.90 | 0.38 |
| KEY 36 | 0.97 | 0.32 | 0.98 | 0.34 | 0.98 | 0.37 | 0.98 | 0.36 | 0.98 | 0.38 | 0.98 | 0.37 |
| CPYDS-10 | 0.93 | 0.56 | 0.93 | 0.59 | 0.94 | 0.61 | 0.94 | 0.61 | 0.94 | 0.62 | 0.94 | 0.62 |
| CPYDS-12 | 0.94 | 0.56 | 0.94 | 0.56 | 0.95 | 0.59 | 0.95 | 0.58 | 0.95 | 0.60 | 0.95 | 0.58 |
| CBC | 0.85 | 0.66 | 0.87 | 0.69 | 0.88 | 0.71 | 0.88 | 0.71 | 0.88 | 0.72 | 0.89 | 0.72 |
| PA | 0.79 | 0.65 | 0.77 | 0.62 | 0.79 | 0.66 | 0.78 | 0.64 | 0.79 | 0.66 | 0.77 | 0.63 |
| GPYDQ | 0.89 | 0.52 | 0.89 | 0.53 | 0.90 | 0.55 | 0.90 | 0.54 | 0.90 | 0.57 | 0.90 | 0.55 |
| PID | 0.83 | 0.72 | 0.84 | 0.73 | 0.85 | 0.75 | 0.85 | 0.74 | 0.86 | 0.76 | 0.86 | 0.76 |
| SA | 0.70 | 0.44 | 0.72 | 0.46 | 0.72 | 0.46 | 0.73 | 0.47 | 0.73 | 0.47 | 0.74 | 0.48 |

[a] Mean interitem correlation.

All parameters were significant ($p < 0.05$).

*Note*: BO: bonding; RE: resilience; SC: social competence; PB: recognition for positive behavior; EC: emotional competence; CC: cognitive competence; BC: behavioral competence; MC: moral competence; SD: self-determination; SE: self-efficacy; CPI: clear and positive identity; BF: beliefs in the future; PI: prosocial involvement; PN: prosocial norms; SP: spirituality; KEY 15: indicator based on 15 key items of the CPYDS; KEY 36: indicator based on 36 key items of the CPYDS; CPYDS-10: 10 subscales of the CPYDS ;CPYDS-12: 12 subscales of the CPYDS; CBC: cognitive-behavioral competencies second-order factor; PA: prosocial attributes second-order factor; GPYDQ: general positive youth development qualities second-order factor; PID: positive identity second-order factor; SA: school adjustment measures.

## *School Adjustment Measures (SA)*

Three items were used to assess the school adjustment of the participants. The first item assessed a respondent's perception of his/her academic performance when compared with schoolmates in the same grade. The respondents were asked to rate "best", "better than usual", "ordinary", "worse than usual", or "worst" in this item. The second item assessed the respondent's satisfaction with his/her academic performance using a five-point response format, i.e., "very satisfied", "satisfied", "average", "dissatisfied", and "very dissatisfied". The final item assessed the respondent's perception of his/her conduct, in which the respondents were asked to rate “very good”, “good”, “average”, “poor”, or “very poor”. Previous research findings showed that these three items and the related scale were temporally stable and valid[19]. Similarly, a higher-scale score indicates a higher level of school adjustment in this study.

## Subjective Outcomes Scale (SOS)

Twenty items were used to assess the participant’s satisfaction with the program and instructor, as well as their perceived benefits of the program at post-tests (i.e., Waves 2, 4, and 6). The response options included “strongly disagree”, “moderately disagree”, “slightly disagree”, “slightly agree”, “moderately agree”, and “strongly agree”. Item 20 (SOS-20) of this scale is “overall speaking, the program was beneficial to my development”. Further analyses were carried out by selecting those experimental participants who found the program to be beneficial based on SOS-20 at Wave 2.

## Data Analytic Strategies

The individual growth curve (IGC) method is an advanced statistical technique that is conducted in order to examine “aggregates” of individual curves rather than separate analysis of each individual trajectory[20]. This method models individual change over time, determines the shape of the growth curves, explores systematic differences in change, and examines the effects of covariates (e.g., treatment) on group differences in the initial status and the rate of growth. A survey of the literature shows that the term “individual growth curve modeling” is commonly used in the field[21,22].

IGC is an appropriate approach when studying individual change as it creates a two-level hierarchical model that nested time within individual[23,24]. The Level 1 model refers to the within-person or intraindividual change model (i.e., repeated measurements over time). It focuses on the individual and describes the developmental changes for each individual (i.e., the variation within individual over time). The Level 1 model estimates the average within-person initial status and rate of change over time. No predictors are included in this model. The basic linear growth model is shown below:

$$Y_{ij} = \beta_{0j} + \beta_{1j}\,(\text{Time}) + e_{ij} \qquad (1)$$

In our study, $\beta_0$ is the initial status (i.e., Wave 1) of the outcome variable for individual *i*. $\beta_1$ is the linear rate of change for individual *i* and $e_{ij}$ is the residual in the outcome variable for individual *i* at Time *t*. $Y_{ij}$ is the value of the outcome variable for an individual *i* at Time *t*.

To test a nonlinear individual growth trajectory across time, other higher-order polynomial trends (i.e., quadratic and cubic slopes) can also be included for model testing. This is shown in Eq. 2, in which *Time* (i.e., the linear slope, $\beta_1$) remains, while $Time^2$ (i.e., quadratic slope, $\beta_2$) and $Time^3$ (i.e., cubic slope, $\beta_3$), are added in the model.

$$Y_{ij} = \beta_{0j} + \beta_{1j}\,(\text{Time}) + \beta_{2j}\,(\text{Time}^2) + \beta_{3j}\,(\text{Time}^3) + e_{ij} \qquad (2)$$

The Level 2 model captures whether the rate of change varies across individuals in a systematic way. The growth parameters (i.e., the within-subjects intercepts and slope) of Level 1 are the outcome variables to be predicted by the between-subjects variables at Level 2. At this level (Eq. 3), an explanatory variable (such as, *group* in the present study) is included to analyze the predictor’s effect on interindividual variation of outcome variable. The errors are assumed to be independent and normally distributed, and the variance is equal across individuals[25].

$$\begin{aligned} Y_{ij} &= \gamma_{0i} + \gamma_{1i}\,(\text{Time}) + \gamma_{2i}\,(\text{Time}^2) + \gamma_{3i}\,(\text{Time}^3) + \gamma_{01}\,(\text{group}) + \\ &\gamma_{11}\,(\text{group X Time}) + \gamma_{21}\,(\text{group X Time}^2) + \gamma_{31}\,(\text{group X Time}^3) + r_{oi} + r_{1i} + \varepsilon_{ij} \end{aligned} \qquad (3)$$

In our study, $Y_{ij}$ is the grand mean for the outcome variable for the whole sample at Time *t*. $\gamma_{0i}$ is the initial status of the outcome variable for the whole sample at Time *t*. $\gamma_{1i}$ is the linear slope of change relating to the outcome variable for the whole sample at Time *t*. $\gamma_{2i}$ is the quadratic slope of change relating to the outcome variable for the whole sample at Time *t*. $\gamma_{3i}$ is the cubic slope of change relating to the outcome variable for the whole sample at Time *t*. $\gamma_{01}$, $\gamma_{11}$, $\gamma_{21}$, and $\gamma_{31}$ are used to test whether the predictor (i.e., *group*) is associated with the initial status, linear growth, quadratic growth, and cubic growth, respectively. $r_{oi}$, $r_{1i}$, and $\varepsilon_{ij}$ are the residual errors that is not explained by Level 2 predictors.

In this study, we tested whether treatment was predictive of students' growth parameters (i.e., initial status, linear change, quadratic change, and cubic change) in several positive youth development indicators across time. In particular, the relationships between these indicators and group were estimated after controlling the effects of gender and initial age. The intercept (i.e., initial status) and linear slope were allowed to vary across individuals.

A dummy variable was created (i.e., *group* — control vs. experimental groups) as a predictor. Participants in the control group were coded as -1 and those in the experimental group as 1. Two covariates (i.e., gender and initial age) were included when examining the predictive program effect on the outcome variables. *Gender* was coded as -1 = male and 1 = female. A similar coding method for a dichotomous variable was found in previous studies[16,23]. For the continuous variables, the grand mean centering method was generally recommended in order to simplify the interpretation of the results[26]. In our study, the mean age was 12. *Initial age* was then centered by subtracting the mean age and, therefore, the centered initial age was generated.

To facilitate the interpretations of the significant interaction effects, we plotted prototypical trajectories as suggested by Singer and Willett[20] in order to demonstrate the effect of treatment on the rate of change across time. The step in creating prototypical plots is generally identical to the method of plotting graphs in regression[27]. For each outcome variable, a linear mixed model (LMM) via SPSS with maximum likelihood (ML) estimation was conducted. As we focused on the entire model (both fixed and random effects), the ML method was used[26]. The procedures for analyzing longitudinal data via SPSS can be seen in Shek and Ma[28].

## RESULTS

Using schools as the units of analysis, results showed that the 19 experimental schools and 24 control schools did not differ in their school characteristics in the aspects of banding (i.e., categorization of students' academic competence), districts, religious affiliation, gender of the students, and source of funding. For the personal characteristics of the participants, results showed that there were no statistically significant differences between the two groups in their sociodemographic background characteristics ($p$ > 0.05 in all cases), except age. The mean age of the control group was higher than that of the experimental group. In other words, the background characteristics of the experimental schools and control schools were highly comparable at Wave 1.

The IGC findings based on several indicators derived from the CPYDS are presented in Table 3. Results showed that there were significant interactions of group and slopes for KEY 15, KEY 36, GPYDS (general positive youth development qualities second-order factor), PID (positive identity second-order factor), PA (prosocial attributes second-order factor), CPYDS-10 (positive youth development based on 10 subscales of the CPYDS), CPYDS-12 (positive youth development based on 12 subscales of the CPYDS), and SA (school academic adjustment).

*KEY 15* — Group was a significant predictor of the linear and quadratic slopes in KEY 15 ($p < 0.05$), but not associated with the initial status and cubic slope ($p > 0.05$) (Table 3). Regarding the linear slope of KEY 15, the control group showed a faster rate of change as compared with the experimental group ($\beta$ = 0.04, SE = 0.02, $p < 0.05$). In terms of quadratic growth, the control group had a slower linear rate of change in the KEY 15 indicator when compared with the experimental group ($\beta$ = -0.04, SE = 0.02, $p < 0.05$).

**TABLE 3**
**Results of Growth Curve Models for Indicators Derived from the CPYDS and School Adjustment Measures**

| | Predictors | | | | | | | |
|---|---|---|---|---|---|---|---|---|
| | Subjects Joining the Tier 1 Program as Experimental Subjects | | | | Subjects Joining the Tier 1 Program Who Regarded the Program as Beneficial | | | |
| | KEY 15 | KEY 36 | PID | SA | PA | GPYDQ | CPYDS-10 | CPYDS-12 |
| Intercept | | | | | | | | |
| Initial status | 4.40** | 156.09** | 4.31** | 3.21** | 4.56** | 4.60** | 4.48** | 4.54** |
| Group | 0.01 | 0.06 | 0.01 | 0.01 | 0.05** | 0.05** | 0.05** | 0.05** |
| Gender | 0.16** | 5.96** | 0.07** | 0.12** | 0.26** | 0.19** | 0.14** | 0.16** |
| Age | -0.03** | -1.69** | -0.08** | -0.09** | -0.06** | -0.04** | -0.03** | -0.04** |
| Linear slope | | | | | | | | |
| Initial status | -0.13** | -5.62** | -0.13** | -0.30** | -0.22** | -0.14** | -0.06* | -0.13** |
| Group | 0.04* | 2.92** | 0.10** | 0.06** | 0.13** | 0.08** | 0.08** | 0.10** |
| Gender | -0.19** | -9.88** | -0.34** | -0.24** | -0.26** | -0.22** | -0.23** | -0.25** |
| Age | 0.05* | 3.19** | 0.11** | 0.15** | 0.06* | 0.06** | 0.05** | 0.06** |
| Quadratic slope | | | | | | | | |
| Initial status | 0.10** | 4.08** | 0.09** | 0.17** | 0.11** | 0.08** | 0.04 | 0.07** |
| Group | -0.04* | -2.38** | -0.08** | -0.04 | -0.11** | -0.06** | -0.07** | -0.08** |
| Gender | 0.10** | 6.12** | 0.23** | 0.15** | 0.17** | 0.12** | 0.14** | 0.15** |
| Age | -0.03 | -2.20** | -0.07** | -0.10** | -0.03 | -0.03 | -0.04 | -0.03 |
| Cubic slope | | | | | | | | |
| Initial status | -0.02** | -0.76** | -0.02* | -0.03** | -0.02* | -0.01* | -0.01 | -0.01* |
| Group | 0.01 | 0.53** | 0.02** | 0.01 | 0.02** | 0.01** | 0.01** | 0.02** |
| Gender | -0.02 | -1.16** | -0.05** | -0.03** | -0.03** | -0.02* | -0.03** | -0.03** |
| Age | -0.01 | 0.45* | 0.01* | 0.02** | 0.00 | 0.01 | 0.01 | 0.01 |

*Note*: KEY 15 = indicator based on 15 key items of the CPYDS; KEY 36 = indicator based on 36 key items of the CPYDS; PID = positive identity second-order factor; SA = school adjustment measures; PA = prosocial attributes second-order factor; GPYDQ = general positive youth development qualities second-order factor; CPYDS-10 = 10 subscales of the CPYDS; CPYDS-12 = 12 subscales of the CPYDS.

$p < 0.05$*, $p < 0.01$**

These results reveal that both groups had similar initial status at the beginning. However, the control group dropped faster and decelerated slower than the experimental group (see Fig. 1).

*KEY 36* — Results indicated that group significantly predicted the linear, quadratic, and cubic slopes of KEY 36 ($p < 0.01$). The test of group difference in initial status of KEY 36 was not significant ($p > 0.05$). Consistent with the results of KEY 15, the control group showed a faster rate of linear change (β = 2.92, SE = 0.70, $p < 0.01$) and a slower rate of deceleration (β = -2.38, SE = 0.65, $p < 0.01$) as compared with the experimental group. Furthermore, a steeper cubic slope was found in the control group, but not in the experimental group (β = 0.53, SE = 0.16, $p < 0.01$). This indicated that the initial status was similar for control and experimental groups. However, the gap between the groups was bigger over time (see Fig. 2).

*PID* — The trend in KEY 36 was also shown in PID. The interactions of group and PID were significant ($p < 0.01$) in all growth parameters (i.e., linear, quadratic, and cubic slopes), except in the initial status ($p > 0.05$). Compared to the experimental group, the control group declined more rapidly (linear slope: β = 0.10, SE = 0.03, $p < 0.01$; cubic slope: β = 0.02, SE = 0.01, $p < 0.01$) and decelerated more slowly (β = -0.08, SE = 0.02, $p < 0.01$). These findings further supported the beneficial treatment effect on participants' perceptions of positive identity over time (see Fig. 3).

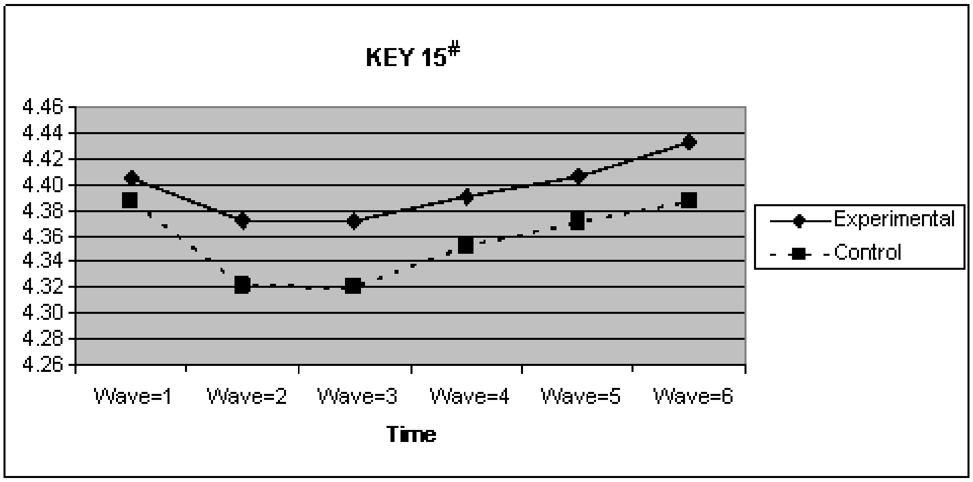

**FIGURE 1.** Growth trajectories of the experimental participants and control participants using KEY 15# as an outcome indicator. #15 key items of the Chinese Positive Youth Development Scale.

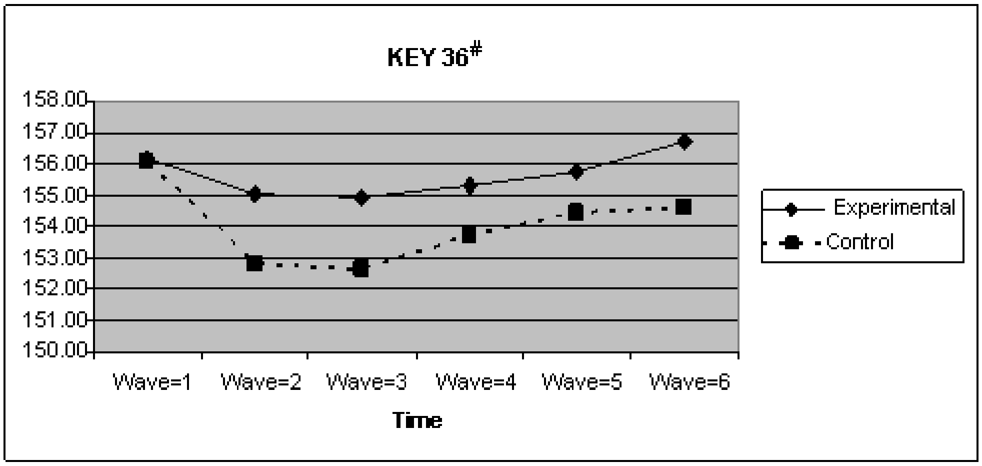

**FIGURE 2.** Growth trajectories of the experimental participants and control participants using KEY 36# as an outcome indicator. #36 key items of the Chinese Positive Youth Development Scale.

*SA* — Lastly, a mixed model was used to test the effect of treatment on school adjustment performance. Group was significantly associated with linear growth ($p < 0.01$), but was not related with other growth parameters ($p > 0.05$). The significant positive linear slope ($\beta = 0.06$, SE = 0.02, $p < 0.01$) indicated that the control group had a steeper initial decline in SA than the experimental group. However, such differences became smaller over time (see Fig. 4).

The positive treatment effects were further supported by comparing the control participants and experimental participants who found the program to be beneficial (i.e., response to SOS-20 in the positive direction). Four significant interactions with group and slopes were found in the four indicators (i.e., CPYDS-10, CPYDS-12, PA, and GPYDQ). In particular, more significant findings were shown in these analyses. Group significantly predicted all growth parameters, including the initial status, linear, quadratic, and cubic slopes ($p < 0.01$). Specifically, the experimental group dropped slower than the

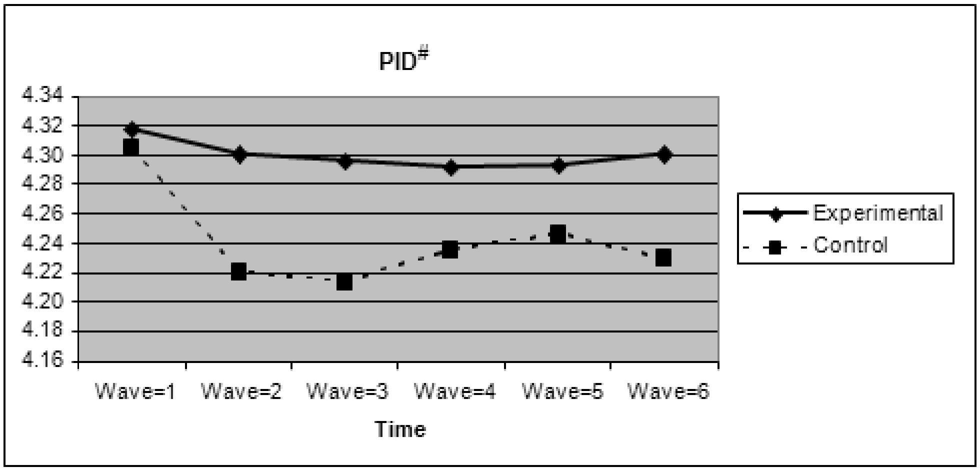

**FIGURE 3.** Growth trajectories of the experimental participants and control participants using PID# as an outcome indicator. #Positive identity second-order factor.

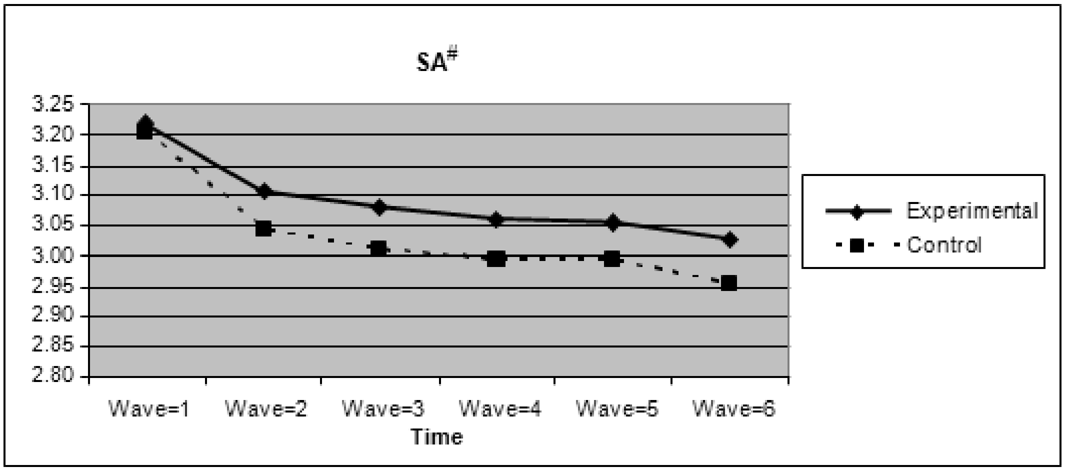

**FIGURE 4.** Growth trajectories of the experimental participants and control participants using SA# as an outcome indicator. #School adjustment measures.

control group in the positive youth indicators as indicated by the positive signs of the linear and cubic slopes (Table 3). This pattern of changes was consistent across indicators. In other words, stable trajectories of positive youth development indicators were found in the experimental group, but not in the control group (see Figs. 5,6,7,8).

## DISCUSSION

The purpose of this paper was to examine the effectiveness of a positive youth development program (Project P.A.T.H.S.) in Hong Kong by using IGC modeling. This is the first known scientific study that adopted a randomized group trial design using longitudinal data to evaluate a positive youth development program in the Chinese context. In addition, other strengths were found in this study. First, the sample size was large and randomly drawn, which could help to generalize the findings. Second, a validated

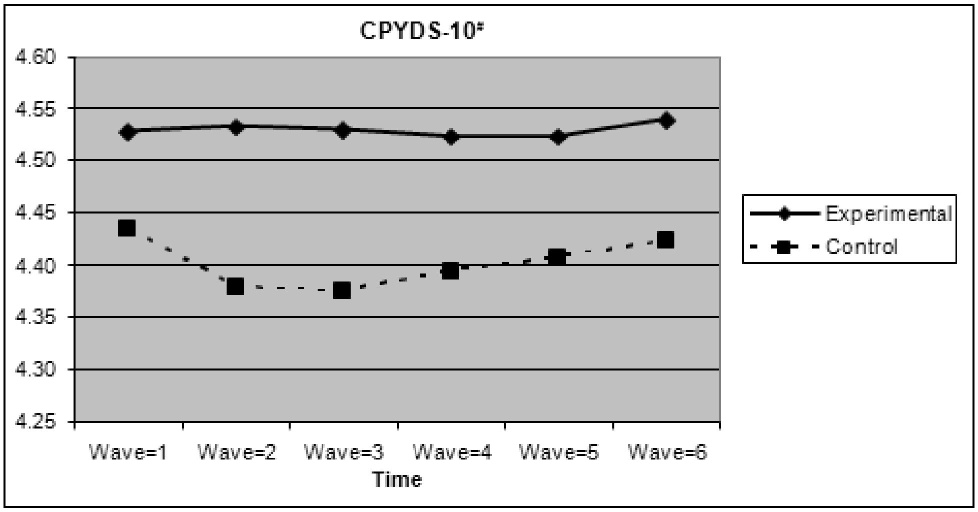

**FIGURE 5.** Growth trajectories of the experimental participants$^{\Delta}$ and control participants using CPYDS-10$^{\#}$ as an outcome indicator. $^{\Delta}$Experimental participants who regarded the program as beneficial. $^{\#}$10 Subscales of the Chinese Positive Youth Development Scale.

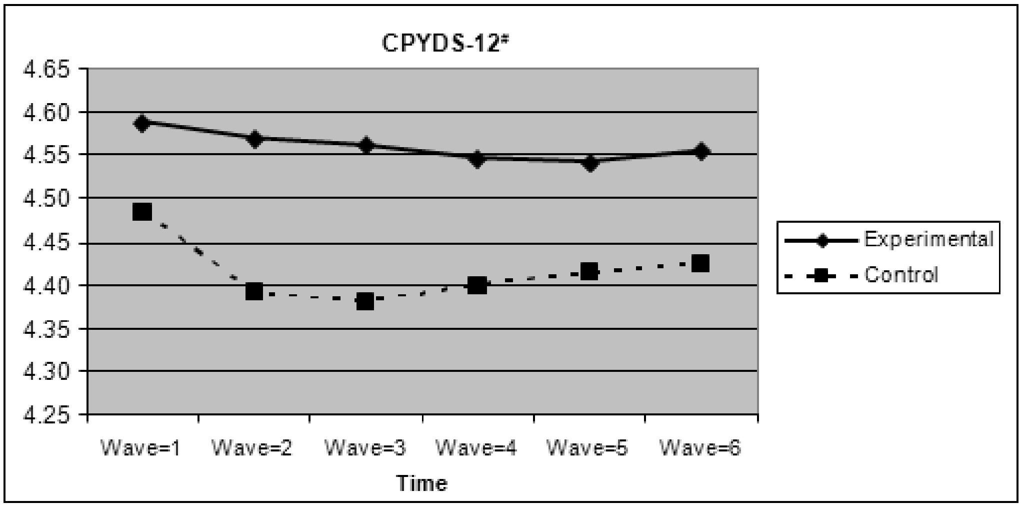

**FIGURE 6.** Growth trajectories of the experimental participants$^{\Delta}$ and control participants using CPYDS-12$^{\#}$ as an outcome indicator. $^{\Delta}$Experimental participants who regarded the program as beneficial. $^{\#}$12 Subscales of the Chinese Positive Youth Development Scale.

measure of positive youth development, the Chinese Positive Youth Development Scale, was used in the study. Finally, IGC modeling, which was superior to generalized linear models, was used in this study.

Compared with the control group, the experimental group generally performed better when various positive youth development indicators were assessed. For example, the findings revealed that experimental participants scored better than the control participants in the areas of psychosocial competencies. In addition, results based on GPYDQ (general positive youth development qualities second-order factor) suggest that the experimental subjects displayed higher scores on eight subscales of the CPYDS (i.e., resilience, social competence, self-efficacy, moral competence, bonding, recognition for positive behavior, spirituality, and emotional competence) than their control counterparts. Furthermore, the

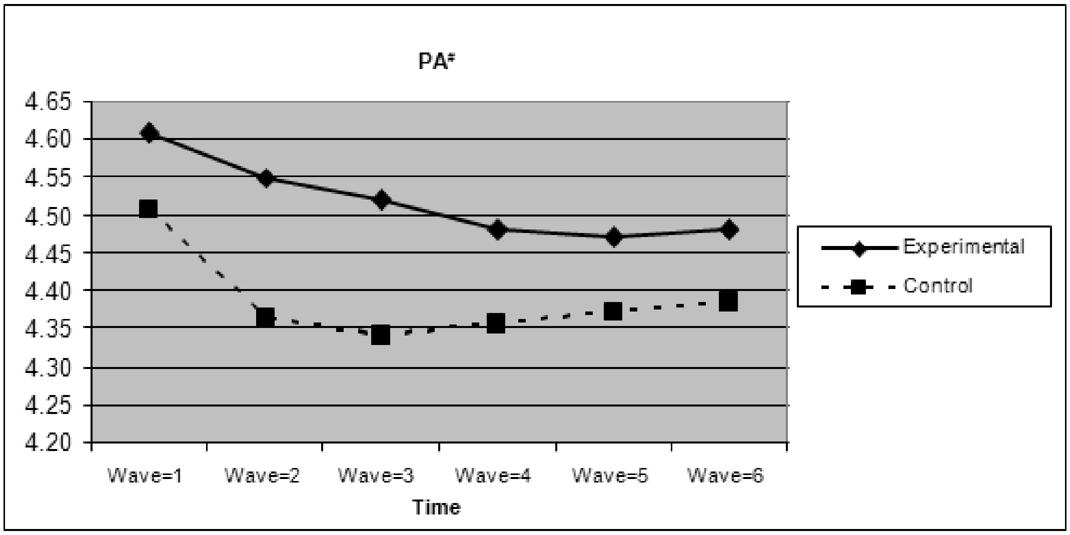

**FIGURE 7.** Growth trajectories of the experimental participants[Δ] and control participants using PA[#] as an outcome indicator. [Δ]Experimental participants who regarded the program as beneficial. [#]Prosocial attributes second-order factor.

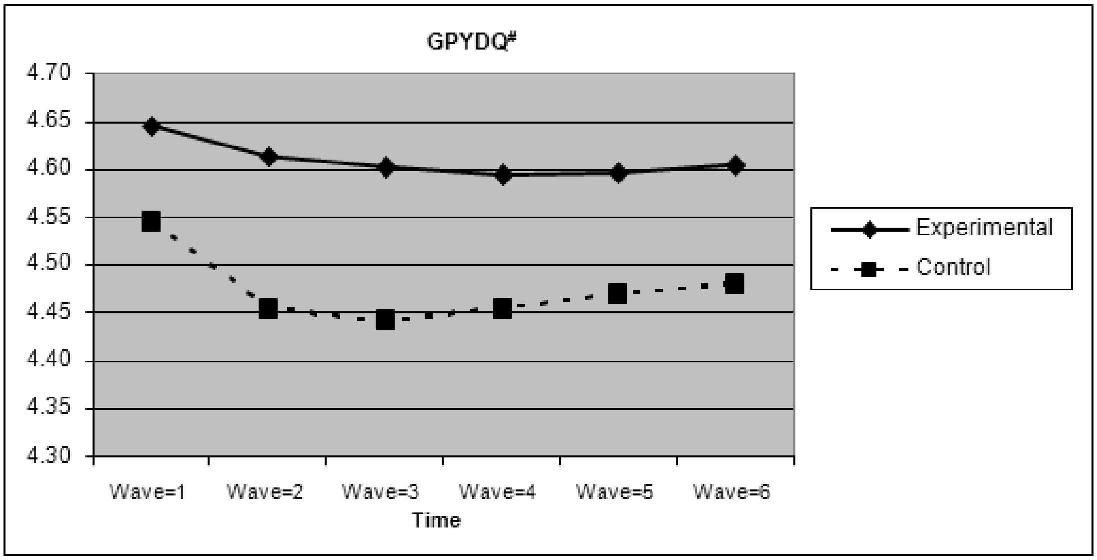

**FIGURE 8.** Growth trajectories of the experimental participants[Δ] and control participants using GPYDQ[#] as an outcome indicator. [Δ]Experimental participants who regarded the program as beneficial. [#]General positive youth development qualities second-order factor.

experimental subjects performed better than the control subjects in PID (positive identity second-order factor, including beliefs in the future and clear and positive identity). Finally, participants from the experimental group had a slower decline in school adjustment than those from the control group. As psychosocial competencies are very important to the holistic development of adolescents, the present findings are encouraging.

Further analyses based on the experimental subjects who found the program to be beneficial to their development (i.e., response to SOS-20 in the positive direction) showed similar, but stronger results. Experimental participants performed better than the control participants in KEY 15 and KEY 36. In particular, the decline in overall positive youth development was slower in the experimental participants than in the control participants in terms of CPYDS-10 (global measure of psychosocial competence and strengths, which includes resilience, social competence, emotional competence, cognitive competence, behavioral competence, moral competence, self-determination, self-efficacy, beliefs about the future, and clear and positive identity) and CPYDS-12 (all subscales excluding behavioral competence, self-determination, and prosocial norms). This suggests that the subjective experience of the participants is paramount. Researchers should examine this factor when examining the effectiveness of adolescent prevention and positive youth development programs.

The above results basically reinforce previous objective outcome evaluation findings based on general linear models[8,29]. In conjunction with previous work using various approaches, such as objective outcome evaluation, subjective outcome evaluation, qualitative evaluation via focus groups, qualitative evaluation via diaries, process evaluation, and interim evaluation[4,5,6,7,8], the existing evaluation findings from the Project P.A.T.H.S. further illustrate the positive impact of the program on youth developmental changes. In view of the paucity of outcome studies in Hong Kong, the present study contributes to evidence-based youth work in Hong Kong[30].

Nevertheless, one interesting observation is that there was a general decline in positive youth developmental attributes across time. While this result is consistent with the finding that adolescent mental health deteriorated across time[31], the decline in “perceived” psychosocial competence is an enigma deserving further investigation. One possibility is that when adolescents mature across time, they have more realistic perceptions about their own development.

## ACKNOWLEDGMENTS

The preparation for this paper and the Project P.A.T.H.S. were financially supported by The Hong Kong Jockey Club Charities Trust.
